# Fijian Veterinarian and Para-Veterinarians' Behavior, Attitude and Knowledge Toward Antimicrobial Use and Antimicrobial Resistance: A Qualitative Study

**DOI:** 10.3389/fvets.2022.898737

**Published:** 2022-06-14

**Authors:** Xavier Khan, Rosemary H. M. Lim, Caroline Rymer, Partha Ray

**Affiliations:** ^1^Department of Animal Sciences, School of Agriculture, Policy and Development, University of Reading, Reading, United Kingdom; ^2^Reading School of Pharmacy, School of Chemistry, Food and Pharmacy, University of Reading, Reading, United Kingdom; ^3^Corporate Engagement, The Nature Conservancy, Arlington, VA, United States

**Keywords:** attitude, knowledge, behavior, veterinarians, para-veterinarians, antimicrobial use, antimicrobial resistance, Fiji

## Abstract

Antimicrobial resistance (AMR) is a global health issue affecting humans and livestock. Reduction in antimicrobial use (AMU) and appropriate use of antimicrobials in livestock production systems have been encouraged. Lack of access to qualified veterinarians, policies regulating AMU and knowledge of AMU and AMR have been identified as drivers of inappropriate AMU behavior in developing countries. Hence, para-veterinarians take a lead role in providing veterinary services to livestock farmers in developing countries. Our previous work found Fijian farmers lack knowledge and understanding of AMU and AMR. However, the attitude, knowledge, and behavior of Fijian veterinary professionals toward AMU and AMR is currently unknown. Therefore, this qualitative study used face-to-face, semi-structured interviews to explore and understand Fijian veterinarian and para-veterinarians' attitude, knowledge, and behavior toward AMU and AMR. A sample of at least ten participants was targeted and recruited from the Central and Western divisions of Viti Levu, Fiji. The Theory of Planned Behavior (TPB) informed the development of the semi-structured interview guide. The interviews were audio-recorded and analyzed using reflexive thematic analysis and deductively using the TPB framework. Our analysis generated three key themes: 1) Antimicrobials prescribed and used based on availability and cost rather than clinical need, 2) Para-veterinarians awareness and knowledge of AMR influence treatment decisions, and 3) Limited resources impede effective consultation and veterinary service delivery. This study demonstrated para-veterinarians (not veterinarians) lacked knowledge and understanding of AMU and AMR. The availability and cost of antimicrobials rather than clinical justification drove antimicrobial prescribing amongst the para-veterinarians. Veterinarians did not visit farms to provide veterinary services; therefore, para-veterinarians provided the veterinary services to the livestock farmers. Lack of human resources, antimicrobials, and physical resources incapacitated veterinary service delivery, where services to farmers' were delayed or not provided at all. Terms of reference for veterinary service delivery and para-veterinarian training framework targeting prescribing, dispensing, use of antimicrobials and risks associated with inappropriate AMU are recommended as part of antimicrobial stewardship (AMS) programmes. Allocation of physical and human resources to Fijian veterinary services should be considered part of AMS programmes to improve veterinary service delivery to livestock farmers and optimize the AMU at the country level.

## Introduction

Antimicrobial resistance (AMR) is a global health issue affecting humans and livestock (1, 2). International organizations such as the World Health Organization (WHO), World Organization of Animal Health (OIE) and Food and Agricultural organization of United Nations (FAO) advocate reduction in antimicrobial use (AMU) and promote appropriate use of antimicrobials in livestock production (1–3). Antimicrobials have been used to mitigate farm biosecurity risks in livestock production systems; however, inappropriate use (growth promotion and prophylactic use) have been reported in developing countries (4–7). A lack of policies regulating antimicrobial prescribing, dispensing and easy access to antimicrobials have been reported as reasons for inappropriate use in developing countries (7–11). The lack of therapeutic guidelines and veterinary legislative frameworks that guide veterinary service professionals have also been reported (5, 9, 11–14). Self-prescribing of antimicrobials by farmers has also been reported in developing countries (6, 12, 15–17). Additionally, in developing countries, investments in animal health and veterinary services have been given less priority compared to human health (2, 13).

Veterinary services in developing countries lack access to qualified veterinarians (18–23). Consequently, para-veterinarians take a lead role in delivering veterinary services, including prescribing antibiotics (5, 9, 11). According to international standards advocated by OIE, only veterinarians (not para-veterinarians) are authorized to prescribe antibiotics (5, 22, 24, 25). Therefore, the prescription of antibiotics by para-veterinarians contradicts international standards (2, 26). However, to combat the growing risks of farmers self-prescribing antimicrobials in livestock farms, para-veterinarians have stepped in to provide veterinary services to farmers to mitigate the risks of uninformed prescribing of antimicrobials (1–3, 6, 12, 15, 18). The OIE competency guidelines mandates veterinarians to receive training and knowledge on antimicrobial prescribing and livestock management (12, 19). The level of training and the legal access to antimicrobials place veterinarians as important guardians of antimicrobials and AMR in livestock production (27). This is contrasted with para-veterinarians where very limited is known about their training and knowledge on AMU and livestock management despite serving as the first line knowledge hubs for livestock farmers (19).

In Fiji, there is limited knowledge about the veterinary services and veterinary professionals apart from the shortage of veterinarians, lack of legislation regulating AMU in livestock production and lack of detailed scope of practice for para-veterinarians particularly relating to prescribing and dispensing of antimicrobials in the Fijian veterinary legislation (current one dates to 1956) (28, 29). Our recent study found Fijian farmers lack knowledge and understanding of AMU and AMR, driving inappropriate AMU (16). Additionally, our study demonstrated that farmers lacked confidence in the local provision of veterinary services, and livestock farmers themselves mitigated farm biosecurity risks including prescribing of antimicrobials (16). However, psychological (attitude and knowledge), including other contextual drivers of AMU and AMR in livestock production from Fijian veterinary services professionals perspectives are unknown (11, 13, 30). Veterinary service professionals are critical partners in livestock production as they interact and provide veterinary services to livestock farmers on daily basis (19, 20, 31). Therefore, the attitude and knowledge of veterinarians and para-veterinarians must be well understood so that antimicrobial stewardship (AMS) programmes can be tailor-made to the Fijian livestock production systems enabling more effective policy implementation (31, 32). Behavioral frameworks such as Theory of Reasoned Action (TRA) (33), Health Belief Model (HBM) (34) and Theory of Planned Behavior (TPB) (33, 35) have been used as socio-psychological frameworks to explore and understand people's behavior. More specifically, several studies using the TPB have demonstrated that veterinarians and para-veterinarians attitude and knowledge influence their AMU behavior (36–38). TPB can help to explore and understand the motivations and barriers of AMU behavior in the context of prescribing, dispensing, and administering (39, 40).

Therefore, this study aimed to explore and understand the behavior, attitude and knowledge of Fijian veterinarians and para-veterinarians toward AMU and AMR in the Central and Western division of Viti Levu, Fiji.

## Methods

### Study Design

A qualitative research design using face to face one-to-one, semi-structured interviews were used. An interpretative epistemological position was taken to analyse the accounts from para-veterinarians and veterinarians (41). AMU in the context of prescribing, dispensing and use in livestock production systems was explored.

### Participants, Recruitment, and Setting

We contacted and recruited para-veterinarians and veterinarians who worked in the livestock production systems located in the Central and Western division of Viti Levu, Fiji. The Central and Western divisions were selected because most Fijians lived and raised livestock in Viti Levu (42). Participants were recruited based on the inclusion criteria in Box 1. We aimed to recruit at least 10 participants to provide in-depth information on their experiences on AMU and AMR. Given that there is no ideal sample size for qualitative studies (43, 44), we presumed at least 10 participants would provide in-depth information to address our study aims. Purposive and snowball sampling methods were used to recruit participants. Our key contacts, the principal agricultural officers in Central and Western divisions, identified potential participants. XK contacted potential participants via telephone and introduced the study to them. XK visited all participants who agreed to participate in a face-to-face interview at a location of their choosing, usually at their clinic or field offices. XK provided the participants with the participant information sheet and obtained verbal consent before the interview. No participant had any prior relationship with XK.

Box 1 **Participant recruitment criteria.**

- Located in the Central Division or Western Division of Fiji
- From Naitasiri, Namosi, Rewa, Serua, Tailevu, Ba, Nadroga-Navosa or Ra province
- Located on the mainland of Viti Levu.
- Location was accessible by road.
- Over 18 years old age.
- A practicing veterinarian or para-veterinarian
- Actively working or had worked in cattle and poultry sector
- Worked in government or non-government organizations
- Experience in the veterinary extension services
- Experience in prescribing, dispensing and administration of antimicrobials

### The Interview

TPB informed the development of the semi-structured interview guide (33, 35). The interview guide in Box 2 was piloted with one participant, and minor modification were made simplify the questions. The interviews were conducted in English at a convenient location to all participants. All interviews were conducted between September and November 2019. All participants were encouraged to speak freely and made aware that XK was interviewing in the capacity of a PhD researcher and was not a veterinarian by profession. An interdisciplinary research team comprising; a male doctoral candidate and pharmacist with experience in agro security, food security and one health (XK), one female academic pharmacist with a doctoral degree in medicine use and safety and extensive experience in qualitative research (RL), a female animal scientist with a doctoral degree and extensive experience in animal sciences (poultry) (CR) and a male academic veterinarian and animal scientist with a doctoral degree with extensive experience in animal sciences (cattle) (PR). XK undertook all the data collection on the study sites. In preparation, XK undertook qualitative methods research training formally via an accredited course and training 'on the job' with RL and her research team that included XK shadowing another researcher conducting interviews, practical guidance on the analysis of data and mock interviews with RL, CR and PR.

Box 2 **Interview topic guide.**

1. Can you tell me about yourself?
*(Prompts: Age, qualifications, years of experience, experience in livestock production? Training? Type of practice?)*
2. Could you describe a typical working day in the clinic/ field?
*(Prompts: what do you do? What type of farmers or complaints do you attend to?)*
3. What do you do when you attend sick animals?
*(Prompts: how and when do you decide to prescribe? Antimicrobials or antibiotics prescribed? How often do you use them? How often do farmers call? How much interaction do you have with farmers? In what stage of animals' sickness, do the farmers ask for intervention? How do you select the antimicrobial or antibiotics? How do you decide on the dose? Availability? Cost? Problems/ challenges faced?)*
4. What do you do when you find the antimicrobials or antibiotics you prescribed on animals is not working?
*(Prompts: Do farmers call back? Consultations undertaken? Check on Dose and Duration farmer used? Consult other Veterinarians or para-veterinarians? Any other medicines used? Usage? Instructions? Medicine substitution?)*
5. Why do you think antimicrobials or antibiotics don't work?
6. *(Prompts: correct dose? The duration? Right medicine? Type of medicine? Stronger medicines? Didn't follow instructions? Medicine not effective? Bacteria being resistant? Antibiotics? antimicrobials?)*
7. Can you tell me what antimicrobial resistance is?
  (*Note: if antimicrobial resistance is unknown*, what is drug resistance? *If not, then* What is antibiotic resistance? *If not, then* what is microorganism resistant to antibiotics? *If not*, what is drug resistance, *(Prompts: if YES: where have you heard from? What do you know about it? View on why medicines don't work? Right dose? The duration? Right medicine? Type of medicine? Stronger medicines? Didn't follow instructions? Medicine not effective? Antibiotics? Antimicrobials? What could be done?*
8. Are there any other comments you want to make about medicine use or antimicrobial resistance?

### Data Management and Analysis

XK transcribed interview recordings verbatim into MS Word and then double checked the correctness of transcriptions against audio recordings. All interview transcripts were anonymised. NVivo 12 was used to analyse the data (QSR International Pty Ltd., UK). XK explored patterns in the dataset, emerging topics, and overarching themes using Braun and Clarke's approach of reflexive thematic analysis (41, 45–47). The data was also deductively analyzed utilizing the TPB informed predetermined topics in Box 3. The analysis process was iterative, involving multiple discussions with the research team, as well as the interpretation of emergent themes in areas of medicine use, livestock production and management. The demographic information was descriptively analyzed and reported. This study was reported using the Consolidated Criteria for Reporting Qualitative research (COREQ) (48).

Box 3 **Topics**

- Attitude toward the AMU
- Social influence (AMU subjective norms)
- Perceived behavioral controls of AMU (Perceived behavioral controls)
- Actual behavioral controls

## Results

### Participant Characteristics

A total of 12 participants were contacted and consented to take part however, only ten were interviewed. Two consented participants became unavailable to take part. The majority of participants were male (*n* = 7, 70%) and between the age of 20–39 years old (*n* = 6, 60%). Over 50% of the participants were from the Western division (*n* = 6, 60%) and from Ba province (*n* = 4, 40%). Most participants had attained a tropical agriculture qualification (*n* = 6, 60%) from a tertiary agriculture University (*n* = 6, 60%) and practiced as para-veterinarians (*n* = 6, 60%). A little below 50% of participants had around 10–20 years of experience in their role (*n* = 4, 40%) and worked in government funded veterinary services departments (*n* = 8, 80%). At the time of study, there were only two veterinarians who were in-charge of the livestock production and veterinary services in the Central and Western divisions of Fiji and both took part in the study. The participants were mostly engaged in providing clinical services from the veterinary clinics and farm advisory services (clinic and fieldwork) (*n* = 5, 50%), and all reported they had training in livestock production (100%) (see Table 1).

**Table 1:** Demographic characteristics of the participants (*n* = 10).

**Category**	**Sub-category**	**N (%)**
**Gender**	Male	7 (70)
	Female	3 (30)
**Age**	20–39years	6 (60)
	40–59years	4 (40)
**Division**	Western	6 (60)
	Central	4 (40)
**Province**	Ba	4 (40)
	Tailevu	2 (20)
	Rewa	1 (10)
	Naitasiri	1 (10)
	Nadroga-Navosa	1 (10)
	Ra	1 (10)
**Level of education**	Tertiary agricultural University	6 (60)
	Tertiary veterinary University	2 (20)
	Vocational agricultural school	2 (20)
**Qualifications**	Tropical agriculture	6 (60)
	Veterinary sciences	2 (20)
	Vocational agricultural	2 (20)
**Occupation**	Para-veterinarian	6 (60)
	Veterinarian	2 (20)
	Para-veterinarian/manager	1 (10)
	Para-veterinarian/Feed mixer	1 (10)
**Years of experience**	0–5years	2 (20)
	5–10years	3 (30)
	10–20years	4 (40)
	Over 20 years	1 (10)
**Type of operations/ Business model**	Government funded veterinary services	8 (80)
	Business	1 (10)
	Cooperative	1 (10)
**Practice (area of work)** ^*^	Clinic and field	5 (50)
	Clinic, field, and administration	2 (20)
	Field	2 (20)
	Clinic	1 (10)
**Livestock production training**	Yes	10 (100)
	No	-

* *Practice or area of work included providing clinical services from the veterinary clinics (clinic), farm advisory services (field) and operational administrative duties (administration)*.

### Interview Findings

The reflexive thematic analysis enabled the generation of three key themes: 1) Antimicrobials prescribed and used based on availability and cost rather than clinical need, 2) Para-veterinarians awareness and knowledge of AMR influence treatment decisions, and 3) Limited resources impede effective consultation and veterinary service delivery.

#### Theme 1 Antimicrobials Prescribed and Used Based on Availability and Cost Rather Than Clinical Need

All veterinary clinic and farm prescribing were done by agriculture assistants or livestock officers who worked as para-veterinarians. Although most of them had attained tropical agriculture qualifications, they said they prescribed whichever antimicrobials were available in their clinic as opposed to following specific prescribing guidelines or making a clear diagnosis before recommending treatment.

“*Yah! There is unavailability of the drugs [and supply is] very [poor]”. Para-veterinarian 3*

“*Well! We only got two kinds of antibiotics in the clinic. [At the moment] we got properacillin [which] we only use now. We [have] not [been] supplied with the Norocillin, so we [use] only [what we have]. We [prescribe antibiotics] based on the supply, give what is available [at that] particular [point in] time. For example, if I have [short acting penicillin's] SA and [long-acting penicillin's] LA so I will use the SA first, so if there is no SA available, I will use the one we have” Para-veterinarian 7*

The participants also shared that there was an inconsistent supply of antimicrobials and there were instances where they were aware that the antimicrobials prescribed were not indicated for the animal disease being treated but they treated the animals anyway to safeguard any potential losses. A few participants said they used human medicines if they were out of stock of antimicrobials licensed for animals.

“*Yeah! I just remember[ed] of a farmer. There [was] no medications and we used the alternate antibiotic medication that was [an] injection, and not used normally to treat those kinds of animals” Para-veterinarian 2*

“*Um! [The] clinic ran short of the antiseptic powder, dusting powder and antibiotics. In order to heal the open wound, I went to the pharmacy, the one which human beings use, so I got that [medicine], I advise[d] the farmer, [and the] farmer used it. After a week, he came back [and] said it work[ed]” Para-veterinarian 5*

The veterinarians expressed that the para-veterinarians only consulted them when needed and typically showed pictures taken of diseased animals to obtain advice. Although veterinarians said they were confident in para-veterinarians delivering veterinary services they doubted para-veterinarians' competence with regards to treating and prescribing antimicrobials.

“*Antibiotics use, and resistance is for the extension officers to fully understand when to use and how to use. Otherwise, they will overuse it because [of] lack of knowledge. They do not know what to do, what to use and how to use [it]. They don't know [then] they just pretend they know it, so they inject penicillin anyway anyhow” Veterinarian 2*

Additionally, some para-veterinarian participants also shared that they prescribed medicines based on their experience, and they had not received any formal training in antimicrobial use and prescribing

“*The major challenge that we face in the field is like trainings. When I joined [the] ministry, I leant from my seniors, so I give medicines from my experience” Para-veterinarian 7*

“*Since last year, I have not received any training, the last qualification was my para-veterinary, and I didn't receive any [training] in antimicrobial and using medicines” Para-veterinary 2*

Some participants also expressed that the farmers do not follow instructions given by the para-veterinarians and usually return complaining about the medicines prescribed to treat sick animals not working. A few participants stated that most farmers were unable to estimate livestock weights and so could not calculate the correct dose. They explained that, therefore, farmers made an estimate of the amount of medicine to administer to their animals without considering what was the correct dose (based on animal liveweight. The participants shared that farmers' usually do not give a complete course of treatment to their animals due to the inflated cost of antimicrobials.

“*Yeah! it is common. [The farmers] do not follow instructions. Most of them, they do not follow instructions” Para-veterinarian 6*

“*Whatever we advise farmers, they do not follow it. They do things wrongly, and then they come back blaming us”. Para-veterinarian 3*

The para-veterinarian participants expressed that there were instances where antibiotics were not needed; however, due to farmers' persistence, participants administered antibiotics to the animals. The participants also shared that they administered antibiotics due to fear of farmers getting aggressive and complaining to higher authorities about veterinary services rather than consider the risks associated.

“*We are giving antibiotics every time we are going out to the field like I said earlier, if you don't give injections to that animal the farmer will create a fuss, [insist to] at least give one injection” Para-veterinarian 7*

In addition, the veterinarians expressed that procurement hurdles delay the supply of the medicines; therefore, the veterinarians said that advance pre-orders were taken from farmers, and medicines were dispatched to farmers once medicines were imported and made available. The veterinarians expressed that those advance orders of medicines were taken from farmers to prevent complains about shortage of medicines to higher government authorities.

“*Oh! This is where it becomes tricky, because farmers complain right up to the Minister, PM's Office, and we take down their names, the numbers and number of drugs and when it comes in you call them to collect the drugs” Veterinarian 2*

#### Theme 2 Para Veterinarians Awareness and Knowledge of AMR Influence Treatment Decisions

The majority of para-veterinarian participants were unaware of the risks associated with AMU. They could not explain and were unaware of AMR. There veterinarian participants were aware of AMU and AMR; however, some participants particularly the para-veterinarians said that they heard of AMR but were unsure of the mechanism of action. However, prescribing and dispensation of antimicrobials in farms were done by para-veterinarians. The majority of participants expressed they rotated the anthelmintics used in cattle and poultry but shared that antibiotics' remained the same all along. Therefore, the para-veterinarian participants shared they prescribed and dispensed the same antibiotics for all illness all year round. The participants shared that they heard of anthelmintic resistance and the purpose of rotating anthelmintics in cattle and poultry; however, most were unaware of antibiotic resistance. Para-veterinarians consistently administering antibiotics in the field on farmers' request was said to be one of the reasons for antibiotic resistance by one participant. Based on past experience with antibiotic prescribing and dispensing, a para-veterinarian participant said the government can address antibiotic resistance by increasing the range of antibiotics.

“*Antimicrobial resistance! I am not really sure what it is. I don't know” Para-veterinarian 2*

“*I think the antibiotics [are] not working because, from my view, I think [animals are becoming] resistance to the antibiotics. We are giving antibiotics every time we are going out to the field” Para-veterinarian 7*

The veterinarians highlighted that para-veterinarians lacked understanding of antimicrobials and incorrectly treated animals in some cases due to a lack of knowledge.

“*Para-veterinarians here, some actually got experience from past because they were trained by previous old veterinarians but there are some you know lack the knowledge and skills, and sometimes, they make things worse than us. I [have] come across those cases, and I have also received complaints” Veterinarian 2*

The participants (para-veterinarians) shared that they injected antibiotics for mastitis; however, there were varying accounts on how mastitis was treated. Some participants used intramammary units first, while some used injectable penicillin (Norocillin). However, the majority shared that they administered antibiotics as a preventative measure.

“*Antibiotic injection is given as a preventive measure to prevent further infections” Para-veterinarian 4*

“*If the intramammary [is used and it] is has improved [then] there [is] no need [for] a revisit, so we just give antibiotic and then after six milking, farmers can start supplying so for example if we treat a cow with intramammary and then it has improved, so the last thing is we give antibiotic injectable and that's it” Para-veterinarian 3*

Most participants (para-veterinarians and veterinarians) said that treatments decisions were based on their convenience. For instance, if they had to revisit farms, they would use short-acting and long-acting injections in severe cases of infections. The batches of antibiotics received from major stores were short and long; therefore, most participants said they will always use all the short-acting injections first and then the long-acting for the rest of the cases.

“*Mostly [used] for [our] convenience. Yes, because you know long-acting, it's for convenience because you can't just go every day to the farm and inject the same cow, so you know long-acting would last 3 to 5 days might as well just give them LA [long acting] injection and sort of keep in contact [with] the farmer, but you don't have to visit every day” Veterinarian 2*

“*When we receive [stock], we receive short-acting, long-acting and the oxytetracycline but sometimes because we [have] plenty of cases where we use short-acting and long-acting, so it finishes and only thing, we mostly left with the oxytetracycline” Para-veterinarian 6*

Some participants shared that they treated all quarters while some treated only infected quarters of cows. Moreover, some used three intramammary per quarter while some used two per quarter, mainly due to the high costs of the intramammary antibiotics. Interestingly, some injected oxytetracycline injection on day one and day three as they perceived it was better to give oxytetracycline injection. The participants also expressed that injecting antibiotics' was necessary in cases of mastitis due to bacterial infection. A few participants said they used expired antibiotics from the clinics and shared that farmers' also used expired left-over antibiotics.

“*Yeah! [So]if they have mastitis [and] if it is severe, so we prescribe injectables. If it is not that severe, we just prescribe the [intramammary] tubes, the antibiotic tubes; we just insert it in the udder” Para-veterinarian 3*

“*Well, sometimes they have their own expired stock of antibiotics, like intramammary they purchase[d] maybe 4 to 5 years ago. They still keep them in stock, and they use those drugs. And then you also got the para-veterinarians who have the expired drugs in the clinic [who] just give them out to them, which is not the good thing, but it happens. I know it happens” Veterinarian 2*

Most participants shared that they used available oral and injectable antibiotics as the first line of treatment in fields irrespective of the disease. A participant expressed that oxytetracycline and chlortetracycline were used to treat coccidiosis and other bacterial infections in poultry. Participants usually switch to the next available antibiotics once treatment does not work.

“*[The number of tubes] depends [on] how many teats are being affected, and then I have to sometimes take estimate. I usually give two [tubes]” Para-veterinarian 5*

“*Oxymav, CTC and it is used for [the] treatment of coccidiosis and other bacterial diseases [in] the poultry” Para-veterinarian 3*

“*If [drugs] doesn't work, [then] we switch to whatever other cheap drugs available” Para-veterinarian 1*

A few participants expressed risks of not following the withholding period after antibiotics use. However, they expressed that farmers' do not follow the withholding period and continue supplying milk despite being advised not to.

“*[The]large farmers, [with] large [herd]of cows, [for] example, with 100 cows, [the]farmers treat majority of [the cows] on farm. [Farmers] don't separate or mark the cows. We have found sometimes, not sometimes may be many times, [we have] seen farmers [and] the laborers don't [follow the] with[holding] period. They milk the cow[s] and supply the milk to [the] factory. The milk [gets] rejected, and we get feedback from the factory. The farmers complain [to us] about milk rejection” Para-veterinarian 8*

#### Theme 3 Limited Resources Impede Effective Consultation and Veterinary Service Delivery

The majority of the participants shared their role involved managing the government funded veterinary clinics and carrying out farm visits, which involved consultation and livestock production and management advisory services. There were only two divisional veterinarians (one in Central division and one in Western division) who were only consulted by para-veterinarians when they required assistance on animal disease management on the farms. The participants expressed that they could not attend to all farmer calls or complaints due to a lack of workforce, transportation, and time. The participants shared that the high costs of medicines also affected their prescribing decisions and scope of veterinary service delivery.

“*Right now, we are running short of staff and only two staff [unlike] before [we had] four staff in a district. The working activities [are] bigger [than] before. So, if we are unavailable, the farmers also complain to our head offices” Para-veterinarian 4*

“*Most [injections are used] for convenience. It is for convenience because you cannot just go every day to the farm. The cost impacts the decision making also.” Veterinarian 1*

The participants expressed that farmers' usually self-prescribe anthelmintics but only visited and consulted them if they think they needed antibiotics. The participants prescribed and dispensed antibiotics from the clinics based on farmers' own diagnosis of their animals' condition. The participants shared that farmers' requests the quantity of antimicrobials base on what they could afford at that point in time. The participants shared that farmers' only sought veterinarians' advice when previous treatments failed. A few participants expressed that farmers' tend to do their research on the internet before consulting them. The majority of participants shared that farmers' were unaware of the remit of para-veterinary services.

“*So, the farmers can easily access a powder, like antibiotics or you know in feed or water, so usually that's the first thing they do, so they go to the nearby station clinic, and they purchase this from agricultural officers and if it doesn't work and it worsens over time than that's when they start calling us and we normally go and inspect” Veterinarian 2*

“*Um! Yeah! Like mostly we have some farmers and, but they mostly use the home remedies. They check on [the] internet and then ask us.” Para-veterinarian 5*

*Yeah, farmers too! They need to be educated properly especially on para-veterinary [services]” Para-veterinarian 4*

The participants shared that the farmers usually visited them in clinics because they were unable to physically visit the farms; there was a lack of staff to operate the clinic and travel to farms located within a wide geographical distance. Participants therefore provide advice and dispense antimicrobials to farmers in the clinics. The veterinarians highlighted that there were gaps in the overall service delivery, including a lack of diagnostic capabilities. The veterinarians expressed that there was a need for training programmes for para-veterinarians on livestock management including AMU and AMR. Veterinarians also shared that they cannot attend to the farm cases because they are involved in administrative matters rather than clinical work. They also highlighted that the para-veterinarians did not provide information to farmers; instead, they were keen to treat animals than engage farmers and provide extension advice. Additionally, the para-veterinarians expressed that they also lacked knowledge on livestock management and the use of antimicrobials, yet they provided services to livestock farmers.

“*[Para-veterinarians] would require a lot more training. Um! Because to be honest, they are not considered para-veterinarians, they are agricultural assistants, but that is not their official job. They are actually extension officers, and yes, they should be dealing with husbandry and management. They need intensive training [and] a lot of learning, like basic things to advise, the farmer needs what is basic. You know that this animal is sick, just telling the farmer to isolate any sick animals, they do not do that. The basic husbandry advice on the farm is missing” Veterinarian 2*

“*I think [para-veterinarians] like to treat. Yeah! But I think they may give advice, but most of the time, from what I have seen, they do not really. It [is] just like they want to attend and solve the problem without actually advising the farmer this is what you know he needs to do, the big, big picture” Veterinarian 1*

## Discussion

To our knowledge, this is the first study to provide an insight into the attitude and knowledge of Fijian para-veterinarians and veterinarians toward AMU and AMR. Our principal findings were that para-veterinarians prescribed and dispensed antimicrobials based on availability and cost rather than clinical need. The para-veterinarians also prescribed antimicrobials without knowing the risks associated with uninformed AMU. There were limited resources such as trained professionals, resources, and time, affecting the quality of consultations and scope of veterinary service delivery.

The antimicrobials, especially antibiotics may perhaps be more inappropriately used when prescribing by para-veterinarians, compared to antibiotics prescribed by veterinarians. This may be due to their lack of training and understanding as compared to veterinarians (9, 18, 19). Our findings suggest that livestock officers who performed the duties of para-veterinarians lacked knowledge and understanding of AMU and AMR, which is similar to findings demonstrated in other studies in developing countries (5, 12, 14). There are gaps in the veterinary service delivery, which are compounded by factors such as a lack of trained veterinarians and resources required in executing effective service delivery. Hence the livestock officers assume the role of para-veterinarians and fill the gaps, thus playing a fundamental role in livestock production and management. The para-veterinarians were allowed to provide veterinary services although they were unsure of the scope of work particularly relating to prescribing and dispensing of antimicrobials. This was due to lack of veterinarians who could provide veterinary services to livestock farmers. Our findings are similar regarding gaps in veterinary service delivery in developing countries, as demonstrated in other studies (7–11).

As compared to developed countries, veterinary access, and delivery in developing countries such as Fiji is limited (19). Therefore, veterinary services delivered by para-veterinarians may ease the workload of the Fijian veterinarians', but the uninformed advice and service provided by para-veterinarians may inadvertently compound AMR issues in the livestock farms, as also demonstrated in other studies (21–23). Taken together, the findings of this and the previous study that focused on Fijian farmers' practice are of grave concern; not only do farmers use antimicrobials inappropriately, the prescribing and dispensing of antimicrobials were also found to be inappropriate. The lack of adequate numbers of personnel meant that para-veterinarians and veterinarians were unable to visit farms. This situation is of concern because the reliance was on farmers' own diagnosis of their sick animals and fulfilments of possibly inappropriate requests for specific types of antimicrobials. There is therefore a greater chance of incorrect clinical diagnosis and use of antimicrobials when compared to clinical diagnosis and treatment by a veterinarian in the field (30). The current approach could set a precedence that self-diagnosis and self-prescribing of antimicrobials by Fijian livestock farmers was acceptable and the norm in everyday livestock production and management despite findings from our previous study demonstrating that farmers lacked knowledge and understanding of AMU and AMR (16). These practices may further aggravate and create obstacles for the implementation of behavioral interventions to safeguard AMU. Therefore, more awareness is required amongst Fijian farmers and para-veterinarians regarding the use of antimicrobials and risks associated with AMU.

Similar to results reported in other studies, our study revealed that some livestock farmers placed pressure (such as threats of reports to government authorities) and heavily influenced para-veterinarians' farm biosecurity risk management strategy such as prescribing and dispensing of antimicrobials (49, 50). In the absence of clinical guidelines (51, 52), para-veterinarians were subjected to a compromised position leading to prescribing antimicrobials even though they may not be indicated, which have been similarly reported in other studies (4–7, 23, 50). We believe the para-veterinarians, including veterinarians, should always practice and provide veterinary services, including prescribing and dispensing antimicrobials, without intimidation and in the most transparent manner (23). The OIE advocates transparency in the management of animal health diseases and veterinary services (18); therefore, we suggest awareness programmes educating all critical Fijian stakeholders in the agri-food value chain on the roles of each stakeholder on prescribing, dispensing and using antimicrobials in livestock production systems. Also, there is a need to develop terms of reference so that para-veterinarians and veterinarians can execute veterinary services without fear (19, 23). All decisions made about livestock management should also consider animal welfare issues where animals should not be exposed to antimicrobials unnecessarily (18).

The availability of antimicrobials further exacerbates the situation as there are greater chances of inappropriate prescribing due to gaps in the supply chain (13, 53). Our results indicate that para-veterinarians prescribed antimicrobials based on what is available rather than triaging and consulting veterinarians. The para-veterinarians also prescribed the antimicrobials, not following any clinical guidelines. Given that there are different classes and dosage forms of antibiotics that can be used to treat mastitis, there was no clinically agreed approach to treat mastitis. The single-use full syringe is used per infected quarter every 12 h to treat mastitis (49); however, our results suggest that either half syringes were used, or the entire course of antibiotics was not completed. Our results also indicated that antibiotics such as tetracyclines were used to treat coccidiosis when contraindicated for the treatment of non-bacterial infections (54). We believe the para-veterinarians were unaware of the indications and contraindications; therefore, they prescribed inappropriately based on what was available at the time of treatment and their experience, which has been similarly reported in other studies (6, 12, 15).

Interestingly, the para-veterinarians were open about their lack of awareness of AMR. Therefore, they continued prescribing and dispensing as they perceived no issues in prescribing antibiotics. Moreover, the para-veterinarians perceived the services they provided complied with best practices and provided services to livestock farmers to avoid complaints that may jeopardize their job. We presume the fear and reticence to challenge and clarify potentially inappropriate practices may be compounded based on age and cultural difference, which was not explored in this present study. Therefore, we suggest further similar qualitative studies exploring other drivers of AMU such as socio-economic, demographic, and cultural contexts.

This present study and our earlier study also alluded to the supply chain issue related to medicines where only a limited range of antimicrobials was available for use (16). The findings are consistent with those reported by Dione et al. (55) in Uganda where supply chain constrains were identified as potential drivers of inappropriate AMU (55). Therefore, we suggest a critical review of the procurement processes so that an informed forecast and procurement of veterinary medicines, including antimicrobials can take place to ensure a sustainable supply of veterinary medicines in the Fijian veterinary clinics. Additionally, the risks of inappropriate may be further mitigated by improving the supply chain of antimicrobials (55). Our finding is similar to results reported in other developing countries where procurement and inconsistent supply of antimicrobials is an ongoing obstacle faced by livestock farmers and veterinary professionals (56, 57). We believe that pharmacists who are experts in medicine inventory management and forecasting may benefit resource-deprived developing countries (56, 58); therefore, consulting and utilizing pharmacist expertise may ease the burdens of procurement. In other developing countries, pharmacists are involved in medicine supply chain management (59). Consequently, we consider adopting a similar approach for the Fijian livestock sector.

Our results suggest that veterinarians were aware of the gaps and inappropriate decisions made by the para-veterinarians in the field, primarily due to the lack of knowledge and training in AMU and AMR. But the para-veterinarians were allowed to continue to be engaged in services delivery because the veterinarians were unable to provide the range of services due to there being only 2-3 (at the time the study was conducted) local veterinarians engaged in livestock production and management in the entire Fijian government veterinary services. The shortage of veterinarians is a widespread problem in developing countries; therefore, the para-veterinarians fill the gaps (7–11). Our findings on the scope of para-veterinarians practice in the agri-food value chain is similar to other developing countries (13, 18, 19).

There are very limited studies that have explored the attitude and knowledge of para-veterinarians and veterinarians in developing countries; therefore, it was hard to compare our findings with other studies; however, the limited studies demonstrated that generally, there was a lack of knowledge on AMU and AMR amongst the para-veterinarians or livestock officers, yet they executed services out of necessity (11–13, 30).

The access to veterinary services and utilizing the local knowledge is quite similar as other studies have demonstrated that farmers only access para-veterinarians if they perceive that para-veterinarians have better training; therefore, there is a general perception of lack of expertise, knowledge and expectation of compromised serviced delivered by the local para-veterinarians (60). Therefore, farmers opt for information and advice from other sources, which may associate a high chance of inappropriate AMU on farms due to being influenced by non-veterinary experts (16, 30).

Our findings implicate the need for a more collaborative approach amongst farmers, para-veterinarians and veterinarians when designing and implementing AMS programmes, based on a clear understanding of each other's roles in livestock production and management (61, 62). Our results indicate that there is currently a standalone approach where veterinary services, including prescribing, dispensing and use of antimicrobials, are farmer-driven rather than clinically based. Therefore, a complete review of Fijian veterinary services delivery is recommended. The OIE performance of veterinary services technical assistance could be explored to guide efforts to address current gaps in the Fijian veterinary services (18).

In addition, the resource gaps require closer consideration by the government, where more policies and funds need to be allocated to fill the gaps in government veterinary services (13, 62). Increasing the number of veterinarian and para-veterinarians are required so that the veterinarians can take the lead clinical role in the field as opposed to spending most of their time with administrative functions. There is also a need to prioritize and provide all resources to the government veterinary services department so that the veterinarians and para-veterinarians are able to execute services fairly, transparently, and without fear (13, 23). Policies and critical competencies empowering veterinarians and para-veterinarians are essential to implement AMS programmes (9, 18, 19). There are immediate measures required to make rational prescribing and dispensing of antimicrobials in veterinary clinics. Antimicrobials should only be prescribed once there is a need for the use of antimicrobials and not based on farmers' demand, which has also been reported in other studies (14). Therefore, we suggest national and sectoral policies promoting the training of more veterinarians and para-veterinarians aligned to the critical competencies stipulated in OIE frameworks and training frameworks for all livestock officers on AMU and AMR (9, 18, 19).

Given that there were obstacles faced in the government veterinary services, the utilization of resources from the private sector such as those in the commercial farms where there is the ability to recruit qualified veterinarians; therefore, a public-private partnership may be considered a feasible approach, especially in developing countries where availability of veterinarians is limited (11, 13, 21).

Nevertheless, policies need to be implemented to promote antibiotic prescribing with supervision and in consultation with veterinarians only. The para-veterinarians should not be permitted to prescribe and dispense antibiotics without a prescription from a veterinarian. The introduction of basic clinical guidelines, which the para-veterinarians can use as a reference point when executing the veterinary services and livestock management is recommended and all decisions made in fields by the para-veterinarians needs to be made in consultation with the veterinarians.

This current study focused on the para-veterinarians and veterinarians, specifically in the livestock sector. However, understanding the attitude and knowledge of livestock officers in non-government services is also required so a more informed AMS programme could be developed (63). Currently, there is a lack of information on the livestock officers involved in the livestock inspection and abattoir services; therefore, establishing their perspectives on AMU practice is equally important. Understanding the whole agri-food value chain is critically important as it may also help provide essential information needed to develop and implement more targeted AMS programmes. We suggest exploring and establishing dialogues by engaging key actors (such as abattoir meat inspectors, farm gate buyers, commercial processors) in the agri-food value chain to develop constructive discussion as that may generate knowledge that may have been missed in one-to-one semi-structured interviews. Acquainting and sharing knowledge on AMU and AMR of these key actors is equally essential so that critical control points could be implemented in the agri-food value chain that may assist in mitigating risks of inappropriate use of antimicrobials.

## Limitations and Future Research

This is the first study that explored the attitude and knowledge of para-veterinarians and veterinarians in Fijian livestock production. Although our sample was mainly from the government veterinary services and accounts may not be representative of the entire Fijian veterinary services, we believe the insights provide a current view of veterinary practices because a significant proportion of veterinary services are rendered by the Fijian Ministry of Agriculture (42, 64) and most farmers access government veterinary services since it is delivered free of charge. There were only two divisional veterinarians (one in Central and one in Western division) who were in-charge of the livestock production and veterinary services provided by Ministry of Agriculture who were interviewed. Both of these veterinarians were interviewed. There was a total of ten divisional para-veterinarians and eight of them were interviewed. However, all other personnel who indirectly worked with para-veterinarians and participated in livestock extension and veterinary services were inaccessible. We acknowledge further studies are required, including the inclusion of all other key stakeholders, to fully understand the veterinary services and the attitude and knowledge toward AMU and AMR more holistically.

The population of veterinary professionals was small, and we interviewed all the practicing veterinarians and eight of ten para-veterinarians in these districts. The concept of data saturation was therefore not applied in this instance.

There are currently limited published studies exploring the attitude and knowledge of the para-veterinarians as compared to veterinarians (36–38). Para-veterinarians play a fundamental role in the decision-making process and overall farm management in fields, especially in developing countries because of a shortage of veterinarians (19). Implementing training and developing the skillset of para-veterinarians would be a crucial consideration when developing antimicrobial stewardship programmes (20); therefore, future studies are required, including public and private veterinary services, to better understand the veterinary service delivery and overall understanding toward AMU and AMR so that more targeted behavioral intervention policies could be developed and implemented as part of AMS programme (31).

## Conclusion

This study demonstrated the lack of knowledge and understanding of para-veterinarians toward AMU and AMR. The AMU was dictated by availability and not by clinical need. Terms of reference for veterinary service delivery, training framework and awareness programmes need to be implemented to improve awareness on AMU and AMR amongst para-veterinarians as part of AMS programmes. A public-private partnership collaborative approach should be considered to enhance the delivery of veterinary services and implement AMS programmes that promote the appropriate use of antimicrobials. Allocation of physical and human resources needs to be prioritized to improve the Fijian veterinary service delivery to livestock farmers. Future studies exploring drivers of AMU in the agri-food value should be considered for the development of AMS programmes to enhance appropriate use of antimicrobials and reduce AMR risks in the agri-food value chain at the country level.

## Data Availability Statement

The datasets presented in this article are not readily available because to ensure the confidentiality of participants and may contain potentially identifiable information. The data supporting the conclusions of this study will be available by the authors, upon request, to any qualified researcher. Requests to access the datasets should be directed to x.r.s.khan@pgr.reading.ac.uk.

## Ethics Statement

The studies involving human participants were reviewed and approved by the University of Reading's School of Agriculture Policy and Developments Ethical Committee (Ref #: 00772P). The Ethics Committee waived the requirement of written informed consent for participation.

## Author Contributions

XK, RL, CR, and PR conceived and designed the study. XK drafted the interview schedule. XK, RL, CR, and PR contributed to the development and review. RL, CR, and PR provided comments and revisions through several iterations of the manuscript. XK contributed in recruitment, interview, recording and transcription, initial coding of transcripts, higher-order analysis and theme development, and drafted the first manuscript. All authors contributed to the article and approved the submitted version.

## Conflict of Interest

The authors declare that the research was conducted in the absence of any commercial or financial relationships that could be construed as a potential conflict of interest.

## Publisher's Note

All claims expressed in this article are solely those of the authors and do not necessarily represent those of their affiliated organizations, or those of the publisher, the editors and the reviewers. Any product that may be evaluated in this article, or claim that may be made by its manufacturer, is not guaranteed or endorsed by the publisher.
