# Supplementary material for: Fijian Veterinarian and Para-Veterinarians' Behavior, Attitude and Knowledge Toward Antimicrobial Use and Antimicrobial Resistance: A Qualitative Study
Source: Front Vet Sci. 2022 Jun 14;9:898737. doi: 10.3389/fvets.2022.898737 (PMC9237570; doi:10.3389/fvets.2022.898737)
Supplement: Supplementary file 1 [file Table_1.DOCX]

**Supplementary file 1: Coding of semi-structured interview transcripts**

The table below illustrates an extract of the codes and topics for theme.

Different colours were used to represent the codes which lead to the development of the theme. Only codes for the theme are presented in the table.

| **Interview number** | **Transcription** | **Codes** | **Sub-themes** | **Theme** |
| --- | --- | --- | --- | --- |
| Interview 2 | I: Do you consult the veterinarian when you have no medicines?  P: Yes. We sometimes consult our vet. We don’t usually disturb them you know they usually busy. They the one who do all tender things for the medicines and that’s the time we consult, and we see what they say.  I: What do you do when you have no medicines, that is the tubes you mentioned earlier?  P: Yes ...we normally use what we have available and then sometimes we see what the farmers have to say and sometimes what the vet have to say than we prescribe accordingly.  I: What do you do when you have no medicines and how you address the situation?  P: You know its all about price……and what is available…you know  I: How do you deal with farmers when you have no medicines in clinics?  P: Yeah! There were farmers!. You know some farmers! They can be dramatic….. I just remember of a farmer he used, like there was no medications and we use the alternate one that was injection, and not used normally to treat those kinds of animals  I: Could you further elaborate on that?  P: Listen so…sometimes we just have to give the medicine you know…the farmers they want it, and they will make stories…so I just give it.  I: What do you do when you find the medicines you gave or prescribed to the animals is not working?  P: Umm,. you know…funny there was a case, that it’s not working, but that medication that is the only medication for that so if it is not working ..i think farmer don’t give it right. They always worry about the cost you know…. that this particular farmer is here and he is saying that medication is not working on his farm.. that one always complains about cost you…. know…but yes prices are too expensive. We just give what the farmer says because….ummm they are one paying. Most times I cant go to farm, so I listen to farmer you know...they know it.  I: Which medicine was that?  P: Like before, farmers used to buy albenol…nilverm.now we don’t have nilverm available in clinics, its albenol, replacement, so nilverm was umm…light liquid and this is a thick liquid so most farmers still go for nilverm but we but we here in the ministry we advise them that we want to try practise rotational dredging since nilverm..nilverm ..it will make like…for them to be ..you know. Resistant to that medication so we always try and change the medications so that they have rotational drenching, and they don’t build resistant to particular medication, so we advise them you always try this one for this month if it doesn’t work you try again for. Next month or maybe we always ask them what the prescription was, or what was the constitution like. How they prescribe what was the measurement or amount they give to that particular calve or cow or what was the weight. Sometime…we come across farmers are not that educated the small scale farmers and they are not educated and they don’t. they sometimes give wrong less medication they sacred of the disease …scared of the not disease but scared their animals might die with new medicines they are using so we always tell them what is the weight of them ..if you don’t know the weight you just estimate the weight of animals. If it’s a big animal estimate 350 to 450kg.if it’s a small calve you can estimate that its small. Like 100kg or less…. if they are still not clear we always go to their farm and we always assist them. You know…. Since this year I have not received any training, the last qualification was my para-veterinary, and I didn’t receive any in antimicrobial and using medicines…like I do what I can…  I: In that case do you always consult farmers first?  P: Yes farmers! they give it in farms so have to…you know..or else again.  I: How often you come across with this situation where they don’t use the right dose, or the right duration of the medicine?  P: Yes, plenty cases …this one only I think otherwise before when we prescribe the medicine, we tell them what kind of animals they have sheep goat and cattle. We tell them how to prescribe. Give them medications.  I: Are medicines always available?  P: No..the medications are not always available with us….out of stock…out of stock…o/s o/s….  I: In that situation when medications are not available, what steps you take?  P: No. When the medications are not available in my clinic, I always check other clinics if it is available but usually you now just give the farmer what is available. sometimes I advise the farmer if he or she can refer to that clinic and buy bulk and if not if there is no clinics if none of the clinics have the medications than I refer to them to the stores and check if they have the stocks and I will check the farmer …if its serious is the case, if they genuinely need it if the stores doesn’t have medications than I just use what we have man…you know the medications are not always available with us…you it’s like that. | - Substitution of antimicrobials without verification - Procurement processes - Dispensation of antimicrobials in bulk - Nonclinical dispensation and prescribing - Cost of antimicrobials and prescribing and use - High cost of antimicrobials - Shortage of antimicrobials - Antimicrobial prescribing - Shortage affecting prescribing of antimicrobials - Prescribing antimicrobials with clinical need - Prescribing guidelines - Self-prescribing by farmers - Human medicine use | *Prescription of antimicrobials*  *Availability of antimicrobials*  *Cost of antimicrobials*  *Purpose of using antimicrobials* | ***Antimicrobials prescribed and used based on availability and cost rather than clinical need*** |
| Interview 3 | I: What type of complains you usually attend to on the farm?  P: Um. main common one at the moment is like the farmers complain about like late arrival of our drugs and unavailability of drugs especially the dredges, antibiotics. if you take it as a dairy farm, mostly we face issues like they have milk rejection on the farm., milk rejection is due to certain reasons there are certain reasons why they having the milk rejection.  I: So how do you decide which one to use?  P: Normally what we do we use short acting for revisit cases, revisit, and small animals , if it is not that serious we use short acting and if it I serious than we use long acting and then the last one is the oxytet we use.  I: Is it always available in the clinic?  P: when we receive .we receive short acting, long acting and the oxytet but sometimes because we plenty cases where we use short acting and long acting so it finishes and only thing we left mostly left with the oxytet.  I: How do you decide which medicine to prescribe?  P: We just make the decision on the farm because its antibiotics any ways and we give what we have. farmers always complain about cost too.  I: Are the costs reasonable to the farmers?  P: Umm…yah it’s reasonable .but not very reasonable..mostly they can’t afford  I: How do you address the challenges of out-of-stock antibiotics and farmers requests?  P: Yah! the only thing we have is the unavailability of drugs very low. And another thing something when we whatever we advise farmers they do not follow it they do not follow it and then they do things wrongly and then they come back blaming us. We even face that kind of issues with the farmers  I: Do farmers use other medicines?  P: Yes. Like mostly farmers they will want to use mostly the home remedies….so they use any drugs without consulting us, but they will use home remedies which we can’t even stop them from using they are more experienced than us sometimes it works. but yes…sometimes whatever we advise they don’t follow it you know……they do things wrongly and then they come back blaming us…. i get sick at times. |  |  |  |
| Interview 4 | I: What types of complains you attend?  P: Plenty …. complains coming up now. All the farmers need help right now they need help. Complains are looking at commodity, sheep, and beef and drugs. Help for the fencing materials, fencing, medicines .,medicines are very important the dredged,. The dewormer they need that, but I am sorry to say now it’s been out of stock…we can’t meet the demands. Sometimes they are very frustrated and are returning back telling us .they are very frustrated …we just give what drugs we have there.  I: What is the problem? shortage of medicines or the price of medicine?  P: Both problem…drugs are always out and price is high…we sometimes just prescribe like how much what they want….  I: Do you participate in ordering of the drugs?  P: Everything we do here, we sell drugs, we have to submit our report, weekly report monthly report ..monthly report, everything is there we selling drugs, the money goes back , than once we put our order for the clinic, but unfortunately there is nothing form there in stores.  I: What do you think can be done about it?  P: There should be training, refresher course and we can call the awareness and call a meeting for all the farmers its locality… they need to have stocks of drugs. hmmm…. ummm there is slight issues ..but like here in the clinic run short off antiseptic powder, dusting powder in order to heal the open wound so I went to the pharmacy, the one which human beings use so I get that one I advise the farmer so farmer used it after a week he came back he said it is working.  I: what else is causing the out of stock?  P: Umm….yah..too much use because we have to give drugs when they sicks….farmers don’t complete the course…follow the instruction I give just because they ….it expensive…you know….Um! because when it’s out of stock …..Suva, no one else even now we try to contact our nearest neighbouring clinics, but they told us same story it is out of stock and end of the day farmer suffers..it’s always the case..farmer suffers but you know |  |  |  |
| Interview 5 | I: How many tubes do you prescribe?  P: They should get 3 tubes some of them they only buy 1 and they say they will try this one and if it works and if it doesn’t work, they will come back….at times it doesn’t works  I: Why do antibiotics don’t work?  P: I think the problem is for the farmers to understand that if they need animals to be good or mastitis that disease to get well than they have to use the treatment that is given like the 3 tubes some of them they use one and that’s it…but I think sometimes we are finding it that antibiotics is not really effective on some animals. Mostly animals that have been treated so many times with antibiotics we change it…I don’t know the change in drugs around name of the drugs only but the antibiotics is there but some of the animals are not and they getting resistant to antibiotics like it doesn’t work in that so we try other we try other vitamins or we just tell the owner to just to leave the cattle free just to see if anything happen to it and could make the cattle to cow to get better…all that.  I: Do farmers follow instructions always? please tell me more about that.  P: Yah its common…. they don’t follow instructions. most of them they don’t follow instructions…. some farmers they these are the farmers they have lot of problems on their farm. It’s like they come they tell us this is the problem on the farm and they go back they never do what we tell them and then after two days or one or two days they come back and tell the animals about to die but we have given them what is needed to be done. umm…some of them they need us to go with them on the farm we usually go and attend to cases but some of them need us to do it for them like giving drugs, treat some of the wounds, or anything like that so we go but some of the farmers they are they do what we tell them if some of them they don’t do it.  I: Do you usually substitute medicines, like when antibiotics don’t work, do you substitute?  P: The only think we ..we have only few drugs with us ah…like antibiotics we usually run out of antibiotics every month and because they give us in less amount and we work what we inject to we give the animals in less amount these what farmers tell us the animals is sick and they tell us ok I have only this amount of money and you have to inject the animal on that amount but the instructions is no there is amount of drugs to be given to that animals but some farmers are different, they can afford everything want for the animals but for substitution on the drugs we have we have less drugs the only drugs that we have mostly is antibiotics in our clinics those are the drugs we have I think its most of the drugs have antibiotics in it but only the names are different. |  |  |  |
| Interview 6 | I: Can you tell me about your working day?  P: Ok in the clinic, for example in the field eh…it depends on the for case in the field it depend on how much cases I been called for the day from last week I didn’t receive any cases last week was just normal office work clinic work only ….only selling drugs and ..yah that’s it..there is less drugs in the office we can serve the farmers who are after drugs but sometimes we are running out of stocks so we not able to deliver the drugs to the farmers.  I: So, you said you running out of drugs or out of stock, so what is it like ongoing issue or is it a something new you facing ?  P: Well! For example, we only got two kinds of antibiotics in the clinic, now we got properacillin and we not supplied with the Norocillin so like now we only use. How to say that! We give based on the supply, give what is available particular time. For example, if I have SA and LA so I will use the SA first, so if there is no SA available, I will use the one we have. this issue for this year like it’s been ongoing from months for we normally submit our returns first week of every month like supplied by 6th or 5th of every first week of the month so our drugs normally later in the month can be last week of the month so once we are submitting the returns that means most of the drugs are out at that time so for that whole month there are some of the drugs we really requested are not in  I: So, you as a paravet what are the challenges you face on the farm or challenges in executing your job on a daily basis?  P: Ok….The major challenge that we face in the field is lack training. Again, like for me when I joined the ministry, I never attended a para-veterinary training I have …. based in clinic. Like basic one for clinical and I attended the one on food safety and meat hygiene so what I learnt when I joined from my senior staff from that time and the reading materials that we normally use that’s what I am doing and the challenges that we normally face in the clinic like… more knowledge. Some of the farmers keep calling us like they have rapid death on the farm, and they want post-mortem reports and want to know what medicine to give. I can’t do a post-mortem, and I don’t know all that medicine. So, I tell them … have to wait for those kinds of decisions  I: Apart from these issues you have highlighted, are there any other challenges or problems you face?  P: At the moment, the manpower for me its I am …. for us its only two ..like one is manning the clinic and the other one normally sometimes goes out….yes shortage of drugs.  I: How often do you have these sorts of situation where farmers call back and complain that antibiotics not working?  P: Umm….eh…like only like for diarrhoea, for diarrhoea cases, like we been giving scour ban .that’s for diarrhoea. We explain to the farmers the dose rate,, like some of the farmers the come after and still saying that it’s not working so can have another one so I normally ask them if you giving the right amount ..they sometimes they say yah..they give very less because cost of the ..because for 120mls its $10.40 may be they give less because it’s so expensive  I: Why do antibiotics don’t work?  P: Yah may be…We are giving antibiotics every time we are going out to the field like I said earlier, if you don’t give injections to that animal the farmer will create a fuss, at least give one injection |  |  |  |
| Interview 7 | I: How do you select antimicrobials when you prescribing?  P: Umm….again it comes down to I think it’s its. Bit different to overseas here it’s also there is a time factor involved…. the supplies our currently selection of antimicrobials is not that wide ummmm…say usually you just select broad spectrum and narrow it down but again it’s a difficult one to answer ..there is lot of factors involved..yah  I: To what extent cost is involved in this?  P: I think its $3.40 for a mastitis tube but I think that’s about for the cost of it and initially they require like 3 minimum some farmers will kick up the fuss for three so like its $15 my way of convincing them is you spend more like $20 on grog a day ….…so fork out. Most farmers I have visited they bought the tubes,,,,some farmers will just buy only one again it won’t treat the mastitis and they will come back complaining so I have to just tell them like if you take 3 you have a high chance of treating the mastitis but you know….out of stock and procurement….arrgghhh  I: Can you please tell me more about the issue of procurement and out of stock?  P: Argh! The government processes! bureaucracy! So, we can prepare request here and say we need this amount, then it goes to our accounts team that would take at least four weeks. Then it goes to [the] economy and then takes another four weeks by the time that they actually finalise the request, the quotations have expired [then] they throw it back like, getting a quotation and by the time companies not willing to provide and most companies we purchase from only want advance payment, so we order from Australia and New Zealand that’s the one we regularly order from. So yeah, that’s the major hurdle.  I: Can you tell me about the role of para-veterinarians?  P: Para-veterinarians here! Some actually got experience from past because they were trained by previous old veterinarians, but there are some you know lack the knowledge and skills, and sometimes, they make things worse than us. I come across those cases, and I have also received complaints…some just do it right at all…they don’t follow and listen…arrghhh |  |  |  |
| Interview 8 | I: Can you tell me more about the medicine out of stock issue?  P: Oh, very different. You know in Australia they keep to the standards, everything is also available, there resources available, I think for them there Code of conduct is more like, what you say! Um! The way they actually handle their cases, they are much more strict they are here, there is no any form of laws, regulations governing us veterinarians basically you can do whatever we want, and we won’t get penalised for it whereas over there it is very tight. They have veterinary councils, board um! So, the standards have to be kept.  I: What else is causing the out of stock of antibiotics in clinics?  P: A lot of use…. like use…literally… Antibiotics use, and resistance is for the farmers to fully understand when to use and how to use especially the farmers and officers; extension officers, otherwise they will overuse it because lack of knowledge. That’s the only thing if they know what to do, what to use and how to use. They don’t know they just pretend they know it, so they inject penicillin anyway anyhow. Oh yes…. Oh! Um! This is where it becomes tricky, because with me, from farmers that we don’t have the drugs in stock, they will complain right up to the top level, you know, Minister, PM’s Office, and the best we can advise them is that while it’s going to be here in a week or a month next month or two months, and we take down their names, we take down the numbers and number of drugs and when it comes in you call them to collect the drugs |  |  |  |
